# Evolutionary Change in Gut Specification in *Caenorhabditis* Centers on the GATA Factor ELT-3 in an Example of Developmental System Drift

**DOI:** 10.3390/jdb11030032

**Published:** 2023-07-08

**Authors:** Gina Broitman-Maduro, Morris F. Maduro

**Affiliations:** Department of Molecular, Cell, and Systems Biology, University of California-Riverside, Riverside, CA 92521, USA; ginam@ucr.edu

**Keywords:** cell specification, *C. elegans*, intestine, gene network evolution, developmental system drift

## Abstract

Cells in a developing animal embryo become specified by the activation of cell-type-specific gene regulatory networks. The network that specifies the gut in the nematode *Caenorhabditis elegans* has been the subject of study for more than two decades. In this network, the maternal factors SKN-1/Nrf and POP-1/TCF activate a zygotic GATA factor cascade consisting of the regulators MED-1,2 → END-1,3 → ELT-2,7, leading to the specification of the gut in early embryos. Paradoxically, the MED, END, and ELT-7 regulators are present only in species closely related to *C. elegans*, raising the question of how the gut can be specified without them. Recent work found that ELT-3, a GATA factor without an endodermal role in *C. elegans*, acts in a simpler ELT-3 → ELT-2 network to specify gut in more distant species. The simpler ELT-3 → ELT-2 network may thus represent an ancestral pathway. In this review, we describe the elucidation of the gut specification network in *C. elegans* and related species and propose a model by which the more complex network might have formed. Because the evolution of this network occurred without a change in phenotype, it is an example of the phenomenon of Developmental System Drift.

## 1. Introduction

**Figure 1:**
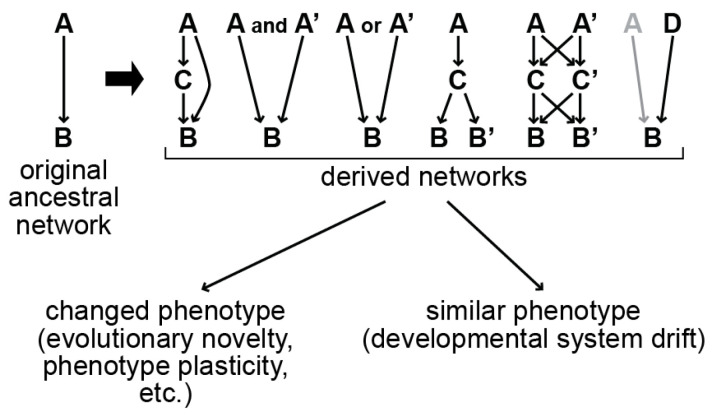
Models for gene network rewiring. We consider a simple gene network in which one factor, A, activates the expression of another factor, B. Networks could evolve through changes in genes by duplication (e.g., gene A giving rise to paralogues A and A’), addition of new regulators (e.g., regulators C and D), changes in *cis*-regulation (new regulatory arrows), and through replacement of factors by others (on the top right, in which regulator D becomes an activator of B followed by loss of regulatory input by A) [14]. Such changes can produce a novel phenotype, but they can also occur without causing any apparent phenotypic change, resulting in Developmental System Drift.

Gene regulatory networks drive early embryonic development in animals [1]. Such networks evolve over time, undergoing changes in *cis*-regulation, duplications, and functional divergence, producing differences in development, or perhaps enabling phenotype plasticity [2,3,4,5]. We consider changes that involve making a simpler network more complex or that rewire major regulatory interactions (Figure 1). A surprising finding has been that changes in gene networks can occur without any apparent change in phenotype, a phenomenon termed Developmental System Drift (DSD) [6]. In these cases, networks undergo distinct evolutionary modifications but nonetheless produce the same phenotype, illustrating that there is more than one way to produce a particular developmental endpoint.

Nematodes of the genus *Caenorhabditis* exhibit examples of DSD [7,8]. We have studied the evolution of an early cell specification event in the nematode *C. elegans* and its relatives in the genus [9,10,11,12,13,14,15,16]. Here, we will review how the intestinal progenitor, E, is specified through the intersection of maternal factors, an extrinsic Wnt signal, and a gene cascade of GATA-type transcription factors. We will describe differences in the network in related species and propose a model for how the more complex network found in *C. elegans* might have evolved through changes in the function of the GATA factor ELT-3.

## 2. The Gut in *C. elegans*

The nematode *C. elegans* has been a model system for developmental studies for nearly a half-century [17]. Its small size, short generation time, and ability to do genetics and genome manipulation have made it possible to decipher genetic mechanisms of development [18]. Early *C. elegans* embryos undergo holoblastic cell divisions in a stereotyped pattern that is nearly invariant from animal to animal [19]. Early cells, or blastomeres, are specified very early; developing cells become progressively restricted in their fate potential by the activity of maternal, then zygotic, factors that drive cell type-specific patterns of gene expression [20,21].

**Figure 2:**
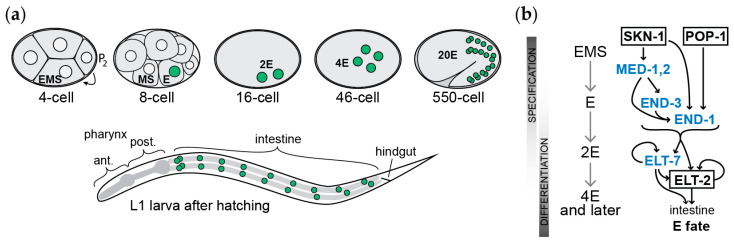
Origin of the gut in *Caenorhabditis* embryogenesis and the core gene specification network. (**a**) Origin of the gut, from the birth of the E cell at the 8-cell stage, through to the 20 cells present in the L1 larva after hatching, a developmental time of 14 h at 25 °C [19]. The nuclei of the E lineage are colored green. (**b**) Sequential activation of transcription factors in gut specification and early differentiation through developmental time. Maternal factors SKN-1 and POP-1 activate a cascade of upstream GATA factors (in blue), ultimately impinging on the activation of the GATA factor ELT-2.

The 8-cell stage blastomere E is the progenitor of the endoderm, which forms the midgut or intestine, as shown in Figure 2a [19]. The specification of E begins before its birth, when the mother cell of E, called EMS, undergoes a cell-cell interaction with its posterior neighbor, P_2_ [22]. After its birth, the E cell undergoes several rounds of mitosis to produce the 20-cell intestine [19]. Even as the E descendants are undergoing mitosis, the gut primordium becomes arranged in a series of morphogenetic events to form an organized tube that will function as the intestine upon hatching [23]. Remarkably, the pattern of cell division of the E lineage can be altered considerably without affecting the morphogenesis of the intestine to form a functional organ [24,25]. Hence, following early specification, gut development can be called regulative, i.e., able to compensate for changes in the numbers of cells despite the relatively fixed number of 20–22 that are observed in the wild type [19,26].

## 3. Elucidation of the Gut Specification Network: Maternal Factors

A summary of the core factors in gut specification appears in Table 1. Early insights into the genetic mechanisms of blastomere specification came from screens for maternal-effect lethal mutations that changed the cell types produced by early blastomeres [27,28]. These identified two major maternal regulators that participate in gut specification. The first, SKN-1, is a bZIP/homeodomain factor that is essential for morphogenesis, pharynx specification, and gut specification most of the time [27,29]. The second, POP-1, is a TCF-like regulator that is essential for preventing specification of gut from MS, the sister cell of E, and which contributes, in parallel with SKN-1, to specification of gut from E [28,30]. Both SKN-1 and POP-1 have functions in other contexts. SKN-1 functions in stress responses and aging, similar to its mammalian orthologue, Nrf [31]. Other screens identified components of a Wnt/MAPK/Src signal that are required for the P2-to-EMS signal that specifies the gut and which act upstream of POP-1 activity [30,32,33,34,35,36,37]. A weak input by the Caudal orthologue PAL-1 can be detected when gut specification is partially compromised [38]. Other weaker regulatory inputs into gut specification have been found that result from more global effects on embryonic gene expression. These include the Sp1 orthologue SPTF-3, the histone deacetylase HDA-1, the p300-like factor CBP-1, the Pur-alpha orthologue PLP-1, the endopeptidase TASP-1, the Prion-like-(Q/N-rich)-domain-bearing protein PQN-82, and other factors that can modulate gene expression epigenetically [39,40,41,42,43]. Finally, other factors have been identified that are important for restricting the activity of SKN-1 to MS and E, such as the RNA-binding proteins MEX-1 and PIE-1 [44,45,46].

## 4. Elucidation of the Gut Specification Network: Zygotic Factors

The zygotic genes that act downstream of these maternal factors were identified through a combination of forward and reverse genetics. The main specifiers of the gut progenitor E are the partially redundant GATA factors END-1 and END-3. Both are transiently expressed in the early E lineage, with *end-3* being activated slightly earlier than *end-1* [13,51,52,53,54]. Both were initially identified through large deficiencies that removed both *end* genes as well as hundreds of other genes, such that the resulting embryos did not hatch [54]. Deletion of only *end-1* and *end-3* results in a fully penetrant loss of gut specification and mostly embryonic arrest, with a small proportion of the embryos hatching as arrested larvae [16]. Instead of producing gut, the E blastomere is transformed into a C-like progenitor, producing ectopic muscle and hypodermis instead of gut [13,24,54]. Single mutants show mild effects. Loss of *end-1* has no detectable phenotype, while loss of *end-3* results in a loss of robust embryonic gut specification, altering the number of gut cells produced by the E cell from 0 to more than 30 [24,55].

The terminal regulator that directs gut fate is the GATA factor ELT-2. It was first identified as a regulator of *ges-1*, a gene encoding a gut-specific esterase that likely functions in digestion [56]. Whereas loss of the *end* genes results in a penetrant loss of all gut, deletion of *elt-2* is compatible with initial gut specification because *ges-1* is still expressed in an *elt-2* null, and animals arrest as larvae with variable amounts of hallmarks of gut differentiation, such as gut granules and luminal tissue [50]. Expression of *elt-2* is initiated by the END factors, and its expression continues throughout adulthood, maintained by positive autoregulation [50,57,58,59]. An adjacent gene to *elt-2*, *elt-4*, was identified that encodes an apparent nonfunctional partial duplication of *elt-2* and will not be discussed further [60].

ELT-2 shares function with another GATA factor, ELT-7, which was identified by its structural similarity to the other endodermal GATA factors [49]. While deletion of *elt-7* has no detectable phenotype, loss of *elt-7* in an *elt-2* null background increases the severity of the gut morphogenesis phenotype apparent in single *elt-2* mutants [49]. Like *elt-2*, *elt-7* is also expressed in the E lineage around the time of E specification and through adulthood.

All of END-1, END-3, ELT-2, and ELT-7 share similar structures as GATA factors and recognize similar HGATAR binding sites [11,58,59,61,62]. Consistent with this, forced individual overexpression throughout the early embryo is sufficient to promote widespread gut specification [13,16,49,50,63]. Forced overexpression of ELT-7 can also reprogram differentiated cells toward a gut fate [64]. This ability is not a widespread property of GATA factors, as overexpression of ELT-1 and ELT-3A, two hypodermis-specific factors, does not promote gut fate [50,65,66,67]. Interestingly, all of *end-1*, *end-3,* and *elt-7* can be replaced with a single multicopy transgene that fuses the *end-1* promoter to the coding region of either *elt-2* or *elt-7* [42,59,65].

The last of the key regulators in gut specification were identified by a search of the genome sequence of *C. elegans* for additional GATA factors, which identified an unusual pair of unlinked, intronless, near-identical coding regions [47]. The two genes, *med-1* and *med-2*, encode divergent GATA factors that were later found to bind a target site with the core sequence RGTATAC, distinct from the canonical HGATAR site [61,68,69]. The *med* factors are expressed in the early E lineage as well as that of its sister cell, MS, and are first transcribed in the mother of both cells, EMS [47,52]. RNAi targeting both genes, or double null mutants, arrest as dead embryos lacking pharynx and muscle (tissues made by MS descendants), and a fraction of the time these also lack gut as well [47,55,70]. The *med* genes are themselves directly activated by SKN-1 [47]. In turn, the MED factors bind the promoters of several genes that are activated in the early MS and E lineages, including *end-1* and *end-3* [68,71].

As shown in Figure 2b, the genes described above can be assembled into a core gene network that describes the specification of gut in *C. elegans* through space and time, from maternal factors (SKN-1 and POP-1), early zygotic factors (MED-1,2), E-specific specification factors (END-1 and END-3), and those that maintain the differentiated state (ELT-2 and ELT-7). The transiently expressed factors upstream of ELT-2,7 can be called primarily specification factors, although even in the absence of ELT-2,7, some features of intestinal differentiation are still apparent in *elt-2,7(-)* larvae [49]. In turn, ELT-2,7, which are expressed throughout the life span and directly regulate terminal genes, are primarily differentiation factors [65,72]. They can contribute to specification, as replacing *end-1*, *end-3*, and *elt-7* with *end-1promoter::ELT-2* or *end-1promoter::ELT-7* transgenes can replace these upstream factors [59,65]. Clearly, there is enough structural similarity among these endodermal GATA factors to permit functional overlap.

Why are there so many factors? The structural similarity among the endodermal GATA factors suggests that functional redundancy contributes to making gut specification robust. Genetic studies have established that the multiple regulators in the network do work this way. Many individual factors can be deleted individually with no apparent phenotypic consequence on gut specification, such as single mutations in *med-1*, *med-2*, *end-1*, or *elt-7* [16,24,49,55]. Deletion of the remaining factors, either alone or in combination to make double mutants, results in a “stochastic” specification: Despite being genetically identical, such embryos no longer robustly specify the gut, so that some embryos make gut and others do not, and those that make gut usually have abnormal numbers of gut cells [24,55]. Examples of such genetic backgrounds include *skn-1* mutants, *end-3* mutants, *med-1; end-3* and *end-1; elt-7* double mutants, and strains engineered to remove the MED-dependent regulatory input into *end-1,3* [13,24,27,55,73]. This probabilistic, binary output of gut specification was found to result from stochastic variation in gene expression of the remaining upstream components, coupled with a threshold of required numbers of transcripts, resulting in a reduced probability of timely activation of *elt-2* [24,74]. In the case of stochastic specification that affects only the E lineage, the gut can still support development to viability. However, in such animals, the intestine is often abnormal in both the number of cells and abnormally increased storage of lipids, presumably indicative of a metabolic defect [24]. Hence, the multiple factors in the network have likely been maintained by selection for both robust development and function of the gut.

## 5. Evolution of Gut Specification in *C. briggsae*

**Figure 3:**
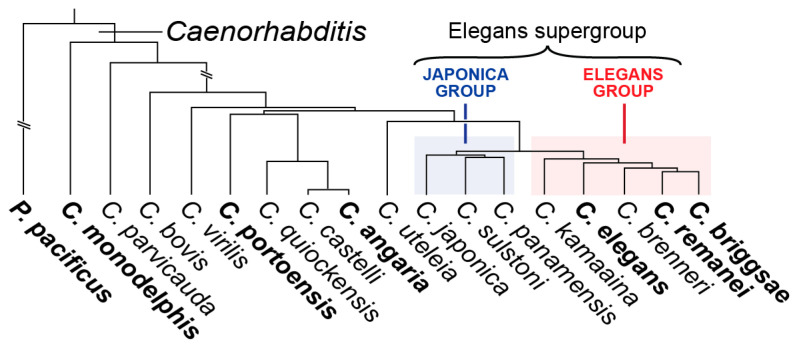
Simplified phylogeny of a subset of species in *Caenorhabditis* from prior publications [75,76,77,84]. The location of *C. virilis* is shown as deduced by the maximum likelihood as reported in [75]. The species referred to in this review are in boldface.

The phylogeny of the *Caenorhabditis* genus consists of more than 50 identified species [75,76,77]. *C. elegans* and its close relatives form the Elegans supergroup of species, which consists of the Elegans and Japonica groups (Figure 3). The nematode *C. briggsae*, a close relative of *C. elegans* within the Elegans group, was the second nematode to have its genome sequenced after *C. elegans* [78,79]. *C. briggsae* is hermaphroditic like *C. elegans*, has nearly the same embryonic development, and is amenable to many methods available in *C. elegans*, including RNA interference, so it has been a useful comparative species [80,81,82,83].

**Figure 4:**
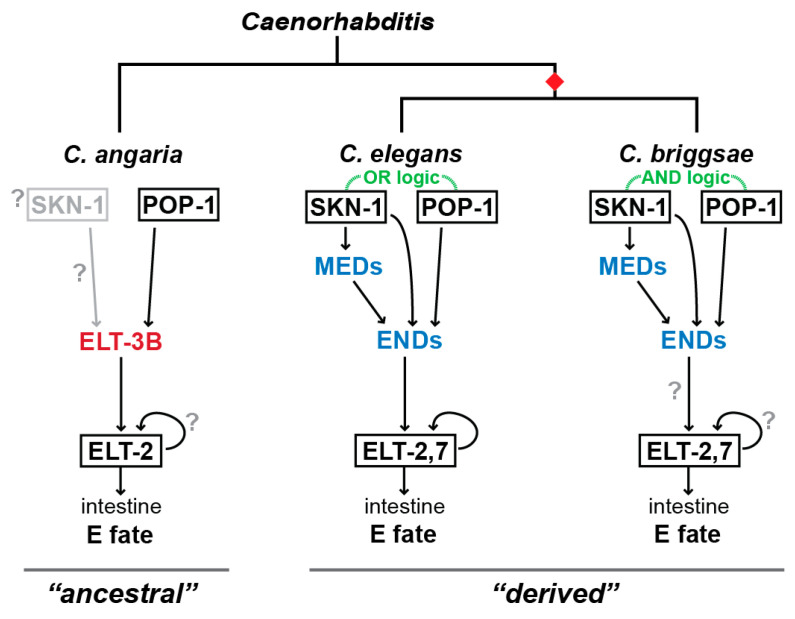
Simplified gut specification network among *C. elegans*, *C. briggsae*, and *C. angaria*. The simpler network of the latter species likely represents the ancestral form of the network in the genus, with the other two representing a derived state that originated at the base of the Elegans supergroup (red diamond).

The zygotic gene network that specifies gut in *C. briggsae* appears to be very similar to that of *C. elegans* (Figure 4). The *C. briggsae* genome contains orthologues of the *med* and *end* genes [10,12,13]. These have similar embryonic expression in *C. briggsae* as their *C. elegans* counterparts, and the *C. briggsae med* genes can rescue *med-1,2* double mutants of *C. elegans* to complete viability [10]. Overexpression of *Cbr-end-3.1* in *C. elegans* can promote widespread gut specification [13]. In *C. briggsae*, RNAi targeting the *C. briggsae end-1* and *end-3* orthologues (the latter of which exists as two nearly identical paralogues, *Cbr-end-3.1* and *Cbr-end-3.2*) results in a penetrant absence of gut, similar to the loss of *C. elegans end-1* and *end-3* [13]. Binding sites for SKN-1 are found upstream of the *C. briggsae med* orthologues, and similarly, MED binding sites are found in the putative regulatory regions of the *C. briggsae end-1* and *end-3* orthologues [15]. Together, these results suggest that gut specification is highly conserved between *C. elegans* and *C. briggsae*. The *med* factors from *C. remanei*, a closely related male-female species, are also similarly expressed in *C. remanei* and able to rescue *med-1,2* mutants of *C. elegans* to viability, suggesting extended conservation throughout the Elegans group [10].

The maternal factors SKN-1 and POP-1 also have clear orthologues in *C. briggsae* [12]. When RNAi experiments were tried with these two factors, surprising differences were obtained. In *C. elegans*, loss of *skn-1* by RNAi or mutation resulted in arrested embryos, but some 20% of these still contain gut, primarily due to input from POP-1 [12,27,30,38]. In the case of *Cbr-skn-1(RNAi)*, arrested embryos were obtained, but these were largely absent of any gut [12]. In *C. elegans*, loss of *pop-1* by RNAi or mutation results in a transformation of MS, the sister cell of E, into an E-like cell, resulting in excess gut [28,38]. However, in *C. briggsae*, the opposite result was obtained. Knockdown of *Cbr-pop-1* by RNAi resulted in the complete absence of the gut and a transformation of the E cell into an MS-like precursor [12,85]. The conclusion was that whereas in *C. elegans*, SKN-1 and POP-1 follow an “OR” type of regulatory logic, where either is sufficient for specification of at least some gut, in *C. briggsae*, these factors follow an ‘AND’ logic, where loss of either factor eliminates gut. This type of change in regulatory logic is an example of DSD [6]. Smaller-scale DSD in gut specification has been found among wild isolates of *C. elegans*, suggesting that the gut specification network has evolved to be robust to these [73].

A change in the promoters of the *C. briggsae end* genes may be responsible for the difference in phenotype of loss of *pop-1* between *C. briggsae* and *C. elegans*. In *C. elegans*, the promoter of *end-1* contains two MED sites, while that of *end-3* contains four MED sites [15,68]. The *C. briggsae* orthologous genes carry only a single apparent MED site in each [15]. When the number of MED sites is reduced in single-copy chromosomal versions of the *C. elegans end* genes, even though gut is specified robustly, depletion of *pop-1* results in the absence of gut [86]. Hence, it is possible to “turn *C. elegans* into *C. briggsae*” with respect to the *pop-1(RNAi)* phenotype by reducing MED regulatory input into *end* expression. Further supporting the importance of MED regulatory input in causing the excess gut in *C. elegans pop-1* mutants, triple mutant *med-1,2; pop-1* embryos also lack gut, showing that in the absence of POP-1, gut specification becomes dependent upon MED input [55]. Taken together, these results strongly suggest that evolutionary changes in the MED binding sites in the *end-1* and *end-3* genes contributed to DSD in gut specification between *C. elegans* and *C. briggsae*.

## 6. Gut Specification Outside of the Elegans Supergroup by the ELT-3 GATA Factor

When high-quality genome sequences became available for additional species in *Caenorhabditis*, it became possible to search for orthologues of the endodermal GATA factors [75]. Within the Elegans supergroup, the MEDs and ENDs were highly conserved, sharing blocks of amino acid conservation both within and outside of their DNA binding domains, as well as in intron/exon structure and putative *cis*-regulatory sites in their upstream flanking regions [15]. A surprising finding was that despite the essentiality of these genes in *C. elegans* and their apparent ubiquity in closely related species, the MED/END factors are absent from the genomes of *Caenorhabditis* species outside of the Elegans supergroup and from more distant nematodes [11,15]. This finding showed that the specification of the gut in these distant species must involve different factors other than MEDs or ENDs. The nature of these was unknown, although it was suggested that perhaps ELT-2 could act as the ancestral endoderm specifier, with the additional factors in *C. elegans* having arisen by a process of repeated duplication and divergence, explaining the structural similarity among the endodermal GATA factors [15,87].

Our recent work in the species *C. angaria*, which lies outside of the Elegans supergroup, revealed that the upstream portion of gut specification in this species consists not of a multifactor GATA cascade but merely of the single GATA factor ELT-3 [9]. In this study, we showed that a *C. angaria elt-2* translational reporter gene is expressed similarly to *C. elegans elt-2* when introduced into *C. elegans* as a transgene. The transgene can functionally complement the larval lethality of an *elt-2; elt-7* double null mutant to viability, showing that it can act in the maintenance of gut differentiation. Consistent with the same role for *elt-2* in *C. angaria*, RNAi targeting *Can-elt-2* resulted in penetrant larval lethality in which the intestine was only partially differentiated, a phenotype similar to loss of *C. elegans elt-2* or loss of both *C. elegans elt-2* and *elt-7* [49,50]. Unexpectedly, the expression of *C. angaria elt-2* in *C. elegans* was completely dependent on END-3 and END-1, despite the absence of genes encoding these factors in the *C. angaria* genome. This suggested that within *C. angaria,* activation of *Can-elt-2* depends on a different GATA factor.

Identification of this GATA factor was straightforward, as only ELT-1, ELT-3, and ELT-5, three primarily hypodermal GATA factors in *C. elegans*, have orthologues in *C. angaria* in addition to the endodermal ELT-2 factor [9,48,88,89]. By examining expression using single-molecule inexpensive FISH (smiFISH), we found that all three show similar embryonic expression to the corresponding genes in *C. elegans*, while *Can-elt-3* showed an additional domain of expression in the early E lineage, from the time of its birth until the 4E stage [9,90]. This pattern overlaps that of *end-3* and *end-1* together, suggesting that ELT-3 fulfills the roles of these factors in *C. angaria* [51,52,74]. Consistent with this, RNAi or a null mutation in *Can-elt-3* resulted in penetrant loss of gut with a phenotype resembling *C. elegans end-1,3* double mutants [9,16].

We tested for the putative roles of SKN-1 and POP-1 in *C. angaria* after identifying their orthologues. Depletion of *Can-pop-1* resulted in a similar phenotype to *C. briggsae pop-1(RNAi)*, namely embryonic arrest and the absence of gut [9,12]. Consistent with a role for Can-POP-1 in the activation of *Can-elt-3*, expression of *Can-elt-3* was abolished in *Can-pop-1(RNAi)* embryos. In contrast, no phenotype for *Can-skn-1(RNAi)* was found, even though transcripts for *Can-skn-1* were successfully depleted by the RNAi treatment [9]. These results suggest that the gut specification network in *C. angaria* is a simpler network in which maternal POP-1 acts upstream of Can-ELT-3, which then activates *Can*-*elt-2* (Figure 4). Consistent with ELT-3 being widespread as the gut specification factor outside of the Elegans supergroup, we found that orthologues of *elt-3* are expressed in the early E lineage in *C. portoensis* and the stem species *C. monodelphis*, the latter of which is considered a basal outgroup for the rest of the known species in the genus [9]. We thus proposed the most parsimonious hypothesis, which is that gut specification in *C. angaria* reflects an “ancestral” mode of gut specification in the genus, while species in the Elegans supergroup, such as *C. elegans* and *C. briggsae*, have a “derived” mode that originated at the base of that group.

## 7. Short and Long Isoforms of ELT-3 Have Different Functions

*C. elegans* ELT-3 was identified by its sequence similarity to other GATA factors [48]. Although the gene was found to be expressed primarily in hypodermal cells, suggesting it may encode a tissue identity factor, a null mutant of *elt-3* showed no apparent developmental phenotype [66]. Forced overexpression of *C. elegans elt-3* throughout early embryos can drive hypodermal specification, but not endoderm specification [50,66]. ELT-3 and the nuclear hormone receptor NHR-25 have been placed in a gene network that drives hypodermal specification downstream of the GATA factor ELT-1 [89,91]. Further studies have shown that *elt-3* is required for gene expression changes, presumably in the hypodermis, that result from exposure of *C. elegans* to oxidative and other environmental stresses [91,92,93,94]. Given that the DNA-binding domains of *C. elegans* and *C. angaria* ELT-3 are identical, the finding that *C. angaria* ELT-3 has a role in the specification of endoderm was therefore unexpected because we also detected *Can-elt-3* in the embryonic hypodermis [9].

**Figure 5:**
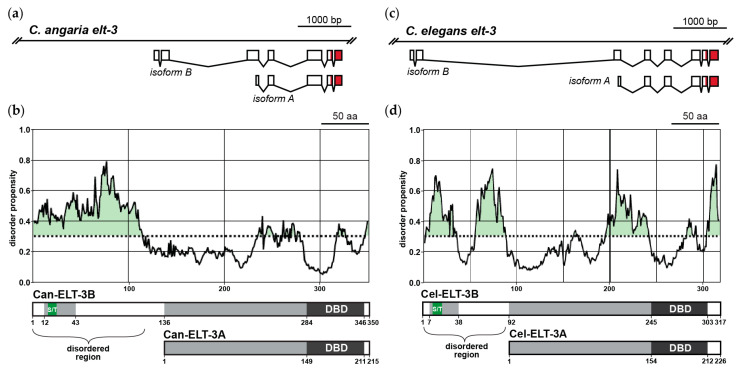
*C. angaria* ELT-3B differs from ELT-3A by having an extended amino region that is intrinsically disordered. (**a**) Diagram showing arrangement of exons in the *C. angaria elt-3* gene and the transcripts corresponding to the B and A isoforms [67]. (**b**) Use of the flDPnn (putative function- and linker-based Disorder Prediction using a deep neural network) algorithm to predict regions of intrinsic disorder in Can-ELT-3B [97]. The results show that the region of highest disorder (with a score of 0.3 or greater) lies in the amino-terminal 118 amino acids, which are within the 135 amino acids specific to the long isoform of Can-ELT-3B [67]. The structures of Can-ELT-3B and Can-ELT-3A are shown beneath the chart, with numbers indicating amino acid positions from the start. The DNA-binding domains (DBD) are shaded dark. Regions of weaker similarity and a serine-threonine region (S/T) are shown after [67]. Similar diagrams are shown for *C. elegans elt-3* in (**c**,**d**).

The apparent paradox was resolved by the finding that *elt-3* encodes two isoforms in both species: A long isoform, ELT-3B, and a short isoform, ELT-3A [9,67,95]. The two isoforms are encoded by differential transcriptional initiation, such that transcripts for both carry the same 3′ end, but the longer isoform has an additional two exons at its 5′ end (Figure 5). Both mRNAs are *trans*-spliced to SL1 [9,95,96]. At the time that *elt-3* was first described, only the shorter ELT-3A isoform was known [48]. In our studies in *C. angaria*, we distinguished expression of the two isoforms using an smiFISH probe that was specific for the longer 5′ sequences present in the mRNA that encodes Can-ELT-3B and found that the long isoform is expressed only in the early E lineage [9]. To test the hypothesis that Can-ELT-3B is endoderm-specific, we forced the overexpression of Can-ELT-3B or Can-ELT-3A throughout *C. elegans* early embryos. Only Can-ELT-3B promoted endoderm, as expected. More recently, we tested whether *C. elegans* ELT-3B could promote endoderm specification when ectopically expressed, and it too has this ability [67].

We further tested whether Can-ELT-3B could rescue gut specification in *C. elegans* when expressed in the correct spatiotemporal context [9]. We constructed a transgene in which Can-ELT-3B::CFP expression is activated by the *end-3* promoter and introduced this transgene into a *C. elegans* strain lacking *end-1*, *end-3,* and *elt-7*. The embryonic lethality of this strain could be rescued by the *end-3promoter::Can-ELT-3B::CFP* transgene. We also confirmed that forced early E lineage expression of *C. elegans* ELT-3B was able to rescue the gut specification of an *end-1, end-3* double mutant [67]. Hence, even though there is no apparent role for *C. elegans elt-3* in endoderm specification, there remains a cryptic ability of the ELT-3B isoform to activate *elt-2*.

As a test of whether the minimal *C. angaria* gut specification network could replace that of *C. elegans*, we introduced both the *end-3promoter::Can-ELT-3B::CFP* and *Can-ELT-2::GFP* transgenes into a quadruple mutant *elt-7 end-1, end-3,* and *elt-2* background, and found that ~50% of animals could be rescued to complete viability [9]. This result shows that Can-ELT-3B and Can-ELT-2 together are indeed sufficient to drive gut specification and differentiation in *C. elegans*, implying that the main zygotic endoderm specification pathway in *C. angaria* consists of only *Can-elt-3B* and *Can-elt-2*.

## 8. How Do ELT-3B and ELT-3A Regulate Different Target Genes?

**Figure 6:**
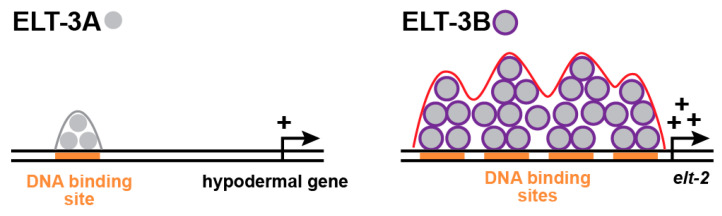
Speculative difference in protein states between the smaller ELT-3A factor and the larger ELT-3B factor. Here, amino-terminal regions of intrinsic disorder favor cooperative interactions with clusters of binding sites in an endodermal target gene like *elt-2*, permitting a more rapid activation of transcription. Modeled after a similar diagram in a prior work [100].

Our recent findings show that ELT-3B, but not ELT-3A, from either *C. angaria* or *C. elegans* can promote endoderm specification in *C. elegans.* The gene and protein structures are similar between the two species (Figure 5A–D). Despite sharing the same DNA-binding domain, these two isoforms must therefore activate different target genes. In humans and mice, the diversity of GATA factor function is often encoded in protein regions upstream of the DNA-binding domains, which mediate protein–protein interactions [98]. If this were true for ELT-3B, we might expect to see a high degree of protein conservation between the ELT-3B-specific parts of Can-ELT-3B and Cel-ELT-3B. However, these regions share only a weakly conserved region that is serine- and threonine-rich, suggesting this portion of ELT-3B may be intrinsically disordered [67]. Using the flDPnn computational tool for disorder prediction, we find that the amino-terminal 118 amino acids of the 135 that are specific to Can-ELT-3B and the amino-terminal 88 amino acids of the 91 that are specific for Cel-ELT-3B are intrinsically disordered (Figure 4D and Figure 5B) [97]. While the amino terminus could be responsible for protein differences due to DNA-binding activity, post-translational modification, stability, or localization, it could be that it is the intrinsic disorder itself that contributes to the ability of ELT-3B to activate endoderm target genes. Intrinsic disorder may allow some transcription factors to become more concentrated at regulatory regions in liquid–liquid phase separations (LLPSs), which could increase the ability of a factor to activate gene expression [99,100]. An intriguing hypothesis, therefore, is that the amino terminus of ELT-3B within *Caenorhabditis* enables an intrinsic change in its state that allows it to interact with multiple dispersed target sites more efficiently (Figure 6). This would mean that ELT-3B-responsive target genes are controlled by “super-enhancers” that permit more rapid initiation of transcription [100]. This might be expected for a factor that has to act within a very short time in the early embryo to drive specification.

## 9. How Did the Gut Specification Network Change in *Caenorhabditis*?

**Figure 7:**
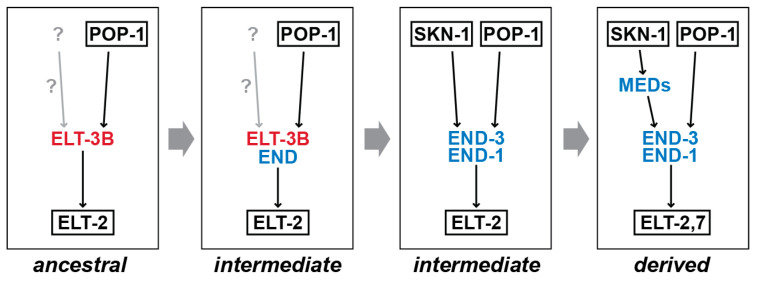
Possible stepwise evolution for the rewiring of endoderm specification within the *Caenorhabditis* genus. Several steps had to occur between the presumed simpler ancestral network and the derived network, and this diagram presents one of many possible patterns of gene duplication and changes in *cis*-regulation. We previously proposed a pattern of network expansion by retrograde evolution, starting from the direct regulation of *elt-2* by a SKN-1 factor [15].

Work in other systems reveals many mechanisms by which gene regulatory networks change by rewiring, although these are usually associated with changes in phenotype (e.g., body plan) or ecology (e.g., life history) [2,101]. In the case of the differences between *C. elegans* and *C. angaria*, there do not appear to be gross differences in either phenotype or ecology, which is typical for Developmental System Drift [6]. Our understanding of gut specification outside of the Elegans supergroup is in its early stages compared to our understanding of the network in *C. elegans*. Results from other species studied so far, *C. briggsae* and *C. remanei* within the Elegans supergroup and *C. angaria*, *C. portoensis*, and *C. monodelphis* outside of it, nonetheless suggest changes that occurred to produce the more complex gut specification network from a simpler network that is likely to have been the ancestral state. Here, we propose a speculative sequence of events that may have led to the derived network in the Elegans supergroup species by predicting one of many possible patterns of gene duplication and divergence and changes in *cis*-regulation, mechanisms that drive DSD [102]. Our inability to be more definitive results mostly from the absence of known “intermediate” species, for example, lacking only one of the MEDs, END-1 or END-3. Either such species have not yet been identified (or had their genomes sequenced), lineages preserving these states are now extinct, or else the changes that must have occurred took place over a relatively short evolutionary timescale. We propose that the network may have evolved mostly from the bottom upwards in a series of steps, as shown in Figure 7. Such a retrograde pattern of evolution has been proposed as a mechanism by which such gene networks arise in general [103].

Of the maternal factors SKN-1 and the Wnt effector POP-1/TCF, we know only that POP-1 has a conserved role in contributing to the activation of gut specification across the genus. In *C. briggsae* and *C. angaria*, knockdown of *pop-1* results in the absence of gut, while in *C. elegans*, simultaneous knockdown of *skn-1* is required to see this contribution, but it is nonetheless there [9,30,38,85]. Since POP-1 acts downstream of the Wnt/β-catenin asymmetry pathway interaction between P_2_ and EMS, it is reasonable to hypothesize that this signal and the regulation of POP-1 are conserved at least throughout the genus [32,36,104].

A role for SKN-1 in gut specification is detectable in both *C. elegans* and *C. briggsae*; however, we could not find a role for the only SKN-1 orthologue in *C. angaria* [9,27,38]. As such, the contribution of SKN-1 to gut specification outside of the Elegans supergroup is not yet known. As SKN-1 directly activates the *med* genes in EMS, some mechanism must act to delay activation of E specification factors like *end-3* until EMS has divided. We previously proposed that this may occur through lower affinity sites in the *end* genes [15]. SKN-1/Nrf has an evolutionarily conserved role in stress response across animals, suggesting that it could have been co-opted into specification late in evolution in *Caenorhabditis* [31]. In an interesting convergence of function, ELT-3 and SKN-1 have a shared role in such responses post-embryonically [92]. If so, regulatory interactions between SKN-1 and *elt-3* could have been co-opted into gut specification.

As mentioned earlier, other maternal regulators such as PAL-1/Caudal and SPTF-3/Sp1 have been found in *C. elegans* that play at least some role in gut specification, as revealed by the ability of knockdowns in these to increase the severity of a gutless phenotype in partially compromised specification backgrounds [38,39]. 

ELT-2 is likely to be conserved as the terminal gut factor, as it is present in most nematode species related to *Caenorhabditis* [11]. Within the genus, the ability of the *C. angaria* gene to replace the *C. elegans* gene confirms that *elt-2* maintains functional *cis*-regulation of its promoter as well as the ability of its product, ELT-2, to activate downstream target genes [9]. Outside of the genus, an *elt-2* orthologue from the vertebrate parasite *H. contortus* can direct gut specification when forcibly overexpressed in *C. elegans*, consistent with widespread conservation [105]. 

The origin of the early, transiently expressed “specifier” gene(s) has been open for speculation for years. Prior work had suggested that the END factors arose as duplicates of *elt-2* [15,87]. This idea was proposed because, until recently, no other GATA factors were known that could specify gut outside of the Elegans supergroup. We now know that ELT-3 is likely to be an ancestral gut specifier, at least in *Caenorhabditis*. Since ELT-3 is structurally similar to END-1 and END-3, which specify gut in both *C. elegans* and *C. briggsae*, it is logical to propose that a prototype *end* gene arose as a duplication of *elt-3*. Indeed, we noted in a prior work that the *end* genes are more similar to *elt-3* than they are to *elt-2* [13]. Because *end-3* and *end-1* orthologues are found within tens of kilobase pairs of each other across many species in the Elegans supergroup, it is likely that the prototype *end* gene underwent duplication to make *end-1* and *end-3* [15]. During this process, the two genes together must have been able to replace the gut-specific function of *elt-3*, resulting in the loss of *elt-3* expression in the early E lineage. The *end* genes maintained their responsiveness to POP-1, as this was already present in *elt-3*. Maternal SKN-1 was co-opted into the specification of MS and E at some point, reinforcing input from POP-1 upstream of the *end*s. Regulatory sites for SKN-1 are still found in the extant *end* genes but could not be found in *Can-elt-3*, suggesting that this regulation arose after *end-1,3* and was not present ancestrally [9,11,15].

The MED GATA factors are found only in the Elegans supergroup and act immediately downstream of SKN-1 in *C. elegans* [15,47]. While MED-1 and MED-2 are not essential for E specification in *C. elegans*, they are essential for specification of tissues derived from MS, the sister of E, and the MEDs appear to activate early E or MS lineage expression of a number of genes [47,70]. Genomic regions containing individual *med* genes from *C. briggsae* or *C. remanei* can functionally replace the loss of *med-1,2* in *C. elegans*, showing they have maintained conservation of expression downstream of SKN-1 and function of the MED gene products [10]. As MED-like factors are not found outside of the Elegans supergroup, these factors likely arose at the base of the supergroup [15]. Exactly when they might have arisen relative to maternal input from SKN-1 is not clear. However, because direct SKN-1 regulation of the *end* genes is genetically detectable in *C. elegans*, we propose that the MEDs arose more recently and became intercalated between SKN-1 and *end-1,3* [15,38]. The same intercalation between SKN-1 and factors that specify MS could have occurred at this time as well [47,70].

Finally, the ELT-7 factor has structural similarity to ELT-3 and plays a reinforcing role in gut development in parallel with END-1 and ELT-2 [49,65,73]. Whether this factor arose later is not known; however, extant ELT-7 orthologues are structurally more like the ENDs in terms of protein size and hence likely to have arisen from an *end* or *elt-3* gene [15]. However, its loss to two species within the Elegans supergroup suggests that it may be dispensable at some level [15]. Other changes in the endoderm network within the Elegans supergroup suggest that the network is highly dynamic in terms of genome evolution. This includes changes in the copy number of the *med* and *end-3* genes, as well as changes in *cis*-regulatory sites [15]. The Elegans supergroup is, curiously, the only clade of the genus in which hermaphroditism is known to have evolved [76]. Indeed, genomic studies across the genus suggest that genome evolution is more rapid in hermaphroditic species [106].

## 10. Examples of DSD in *Caenorhabditis* and Other Animals

Several examples of DSD are known in *Caenorhabditis* and other animals are reviewed elsewhere [7,8]. Whereas the type of DSD we have uncovered in endoderm specification is largely due to changes in transcription factor gene networks, others have been found to involve changes at multiple molecular mechanisms. In one of the defining examples of DSD, the specification of the precursor cells that form the hermaphrodite vulva has been the subject of study in *Caenorhabditis* and related nematodes, including *Pristionchus* [107,108]. In both species, the same set of three hypodermal cells will form the vulva primordium. In *C. elegans*, LIN-3/EGF (Epidermal Growth Factor) signaling from a cell outside the equivalence group, the anchor cell (AC), specifies the primary fate in the closest cell, while the neighboring cells receive less EGF signal and lateral signaling by LIN-12/Notch, which specifies the secondary fate [109]. Among different *Caenorhabditis* species, cryptic variation is observed in how EGF, Notch, and Wnt pathways work cooperatively to ensure robust vulval specification [110]. 

In an example of gene network rewiring in the evolution of vulva specification, a 59-bp *cis*-regulatory module upstream of *lin-3* contains three *cis*-regulatory sites that are required for normal expression in the anchor cell [108]. In *C. angaria*, only one of these sites is present, but it is nonetheless sufficient to drive normal expression of *lin-3* when used as a transgene in *C. elegans*, hypothesized to be due to an unknown factor that can activate that module in both species [108]. We identified conserved putative *cis*-regulatory sites in the *med* and *end* genes across the Elegans supergroup, but comparative functional analyses have not yet been performed [15]. The *elt-2* promoter sequences have been compared among a subset of these species to identify GATA site clusters in *C. elegans* by conservation, but not for cross-species comparative functional studies [58,59]. As multiple redundant factors appear to be at work in endoderm specification, it is likely that some species may show different phenotypes from *C. elegans* when individual factors are knocked out. Similarly, induction of gut by the P_2_-to-EMS cell signal involves Wnt, MAP kinase, and Src tyrosine kinase pathways working in parallel [35,111,112]. Hence, there are opportunities for future work on cryptic variation in endoderm specification in both cell signaling and regulation of gene expression across the genus.

In the more distantly related species, *Pristionchus pacificus*, vulva specification exhibits significant changes in how fates are assigned, despite conservation of the underlying primary and secondary vulval fates with *Caenorhabditis* [107]. In this species, both the anchor cell and the larger somatic gonad provide an inductive signal for the primary fate, while two other cells, the mesoblast M and a more posterior hypodermal cell, induce the secondary fate [107]. This predicts that in more distant species, even larger-scale differences in gut specification may be found compared with those occurring within *Caenorhabditis*. Intriguingly, an expansion of *elt-3*-like genes is found in *Pristionchus*, perhaps hinting at an independent radiation of endodermal GATA factors [11]. This possibility is currently being investigated.

One well-known example of change in a developmental gene network outside of nematodes involves that which determines segment identity in *Drosophila* and related species. Compared with *Drosophila*, the underlying gene network has undergone rapid evolution, particularly in its upstream inputs [113]. In cyclorrhaphan flies, a group that includes *Drosophila*, the maternal anterior specification factor Bicoid arose from a duplication of Hox3 at the base of the group [114,115]. Outside of Cyclorrhapha, different factors appear to play a similar role as *Bicoid* [116,117,118,119]. The replacement of other upstream regulators by *Bicoid* resembles the replacement of ELT-3 by END-1/3 in *C. elegans*. However, whereas early embryos of *Caenorhabditis* are almost indistinguishable from one another in their embryonic development, Cyclorrhapha embryonic development shows a wide variation, for example in the speed of embryogenesis and in the length of the germ-band [81,120,121,122].

Another example of DSD in a gene network is found in tunicate larvae, in the development of *Ciona* compared with that of the distant relative *Molgula* [123]. These two species share similar cell lineages in development, despite hundreds of millions of years since the common ancestor [124]. Within the developing motor ganglion of the two species, expression of several regulatory genes, including the homeobox genes *Dmbx* and *Vsx*, is conserved [123,125]. However, the upstream regulators have undergone a change in expression and regulatory logic. This is revealed in the ability of *Dmbx cis*-regulatory sequences from *Molgula* to drive proper spatiotemporal expression in *Ciona* but not the reverse, implying DSD [123]. The authors proposed that slight differences in developmental time, with *Molgula* development occurring 10% faster, added to the constraint that might be imposed by fixed cell lineages, may have imposed a selection for DSD-type changes to keep neural specification robust [124].

## 11. Additional Questions for Future Work

### 11.1. Gut Specification in Other Species in the Genus

The apparent rapid changes in genes involved in gut specification suggest that there are likely to be other examples of either network rewiring or cryptic changes in the relative contribution of different paralogous genes, especially among species in the Elegans supergroup, for which complex patterns of gene duplication were observed [15]. Although such changes are probably not likely to be as major as the rewiring that expanded *elt-3* and produced several paralogous GATA factors, there could be examples of the gain or loss of specific regulators. Indeed, among the 20 Elegans supergroup species examined, two appeared to lack *elt-7* orthologues [15]. Alternatively, there may be some Elegans supergroup species that have retained expression of *elt-3* in the early E lineage, for example, or have further rewired their gut specification networks by additional duplication and divergence or changes in *cis*-regulation [15]. A further challenge will be to identify genes that have been co-opted into the pathway that are not GATA factors. Single-cell transcriptomics methods have advanced considerably in recent years, which might permit the identification of such factors [52,126]. The gold standard in determining whether factors expressed in the E lineage participate in specification is to delete them by mutation or test for a phenotype by RNAi, as we did for *elt-3* in *C. angaria* and *end-1,3* in *C. elegans* and *C. briggsae*, and show that gut specification is abolished [9,13,16]. Methods in species other than *C. elegans* and *C. briggsae* are less well developed, which may limit such functional studies [127].

### 11.2. Function of ELT-3B in C. Elegans and Other Species in the Elegans Supergroup

The ability of ELT-3B from *C. angaria* and *C. elegans* to activate *elt-2* creates an opportunity to study *cis*-regulation of *elt-2*, as prior work on *elt-2* regulation has considered only the known END-1,3 and ELT-2,7 factors [58,59]. Is the activation of *elt-2* by ELT-3B distinctly different? What microevolutionary changes have occurred in the DNA-binding domains, and how does the amino terminus affect DNA binding, if at all?

There is also the question of what ELT-3B could be doing in *C. elegans.* Its cryptic ability to promote *elt-2* expression and gut specification when forcibly expressed in early embryos, strongly suggests that ELT-3B has been maintained by selection for some other function outside of specification. An analysis of the expression of each isoform in *C. elegans* might suggest what the possible role of ELT-3B might be, as it is likely to be cell-autonomous. Prior work suggested functions for ELT-3 in the adult intestine, although others have ruled out such a function [128,129]. With new results showing that ELT-3B can activate *elt-2*, it remains possible that a function for ELT-3B is conditional, perhaps increasing expression of *elt-2* under stressful conditions [67]. Recent studies on the roles of ELT-3 in stress responses have considered only the effect of alleles that are predicted to affect the DNA-binding domain, which would affect both isoforms [92,93,94].

### 11.3. Evolution of ELT-3 Orthologues and Isoforms

To our knowledge, the dual functions of ELT-3B in endoderm and ELT-3A in ectoderm represent the first identification of a canonical *C. elegans* GATA factor with isoforms that have roles in distinct germ layers [9]. It is not known how widespread the long/short isoforms of *elt-3* genes may be, as this has not yet been examined [11]. The upstream exons in *elt-3B*, being smaller, less conserved, and upstream of a large intron, might make identification by gene prediction algorithms more difficult. A more detailed analysis of *elt-3* genes within and outside of *Caenorhabditis* would reveal whether the long isoform is a recent innovation or more widely conserved, and hence whether ELT-3B as an ancestral gut specification factor is more widespread. Curiously, when it came to the evolution of GATA factors in general, orthologues of *elt-3* appeared to have undergone increases in copy number in several species [11]. In our study on *Can-elt-3*, we found that only one of two *C. monodelphis elt-3* paralogues is expressed in the early E lineage [9]. Therefore, it may be that different functions for *elt-3* genes, in addition to gut specification, could evolve through gene duplication and divergence.

### 11.4. Amenability of Gut Specification to Rewiring

Much of the research in the *C. elegans* gut specification network has been directed at understanding how the network responds to perturbations, such as the removal of regulators by mutation, modification of *cis*-regulatory sites, and replacement of factors by others [9,24,59,67,74,86,87]. In general, these results have shown that the network is highly amenable to being rewired. To perform functional studies of putative networks from other nematode species, *C. elegans* can be used as a test system in which to synthetically replace the network with that of other species, as we did with *C. angaria* [9].

### 11.5. What Caused the Rewiring of Endoderm Specification?

Figure 7 above provides a speculative model for the stepwise conversion of the putative ancestral gut specification network into the extant network in *C. elegans.* However, the selective pressures that might have led to the rewiring of the network are still unresolved. Haag and True [102] considered two mechanisms that drive DSD that are pertinent to the rewiring of a transcriptional factor network. One of these mechanisms is the duplication of an ancestral gene to result in two subfunctionalized paralogues [130]. In endoderm specification, this mechanism accounts for the origin of the *end-1* and *end-3* genes from a prototype *end-1* gene, or *elt-3* itself. Once *end-1* and *end-3* had diversified in function, it would be difficult to lose either of them. Consistent with this, subtle differences in binding affinities of END-1 vs. END-3 on the *elt-2* promoter have been observed [58,59]. Such differences would be consistent with END-1 and END-3 paralogues having co-evolved with subsets of ancestral ELT-3 target genes. The overexpression of ELT-3B in our cross-species studies would likely overcome such differences, so subtle differences in target genes would not be apparent [9]. The driver for the radiation of factors may have been that increased numbers of regulators result in higher robustness to environmental fluctuations or more rapid development. In some insects, for example, the A/P patterning gene networks evolved more complexity through changes in upstream regulators, which may have allowed more robust patterning under pressure for more rapid development [117,121]. It is not clear whether more rapid development could be the driving force in endoderm development with *Caenorhabditis*, although some of the fastest-developing nematodes are found in the clade that includes this genus [131]. The speed of embryogenesis between *C. angaria* and *C. elegans* is about the same, although there are minor differences in particular milestones [132]. It is also not apparent that the simpler network in *C. angaria* is in any way less “robust” than the more complex network in *C. elegans*. Under laboratory conditions, *C. angaria* animals grow well, especially considering that they are a male-female species, which tend to suffer from inbreeding depression when cultured for long periods [127]. We also do not know if there are additional regulators in *C. angaria* that play roles analogous to the MED, END-1, and END-3 factors but that are not GATA factors. These factors could make the gene network more robust in *C. angaria*.

A second mechanism that drives gene networks DSD involves different types of selection acting on the direct targets of a pleiotropic transcription factor [102,133,134]. Here, the factor activates two distinctly different target genes. Directional selection acts on the *cis*-regulation of one of the targets to increase its expression, while the second target undergoes stabilizing selection. This drives changes in the second gene to maintain its expression levels, providing a selection that may ultimately result in the recruitment of a different activator [133]. DSD would thus be observed as a change in regulation of this gene from the ancestral activator. In the case of endoderm specification in *Caenorhabditis*, this would require that the ancestral *elt-3* gene function in two processes. Indeed, *C. elegans* ELT-3 functions in the post-embryonic hypodermis, where it is required to activate genes that respond dynamically to oxidative stress as well as to modulate expression of hypodermal genes involved in cuticle structure [92,93]. We observed expression of *C. angaria elt-3* in the embryonic hypodermis, similar to expression of *elt-3 C. elegans* [9,48]. Hence, it is likely that ancestral ELT-3 activates both *elt-2* in the early endoderm and stress response genes in the hypodermis. Over time, directional selection acting on its role in the hypodermis would drive stabilizing selection on its role in activation of *elt-2*. This could result in the reassignment of gut specification to what became *end-1,3*. Once gut specification was uncoupled from stress response, the genes involved were free to undergo more rapid evolution. Hence, it is possible that both gene duplication/subfunctionalization and pleiotropy of ancestral ELT-3 led to the radiation of gut specification genes in *Caenorhabditis*, driven by directional selection for ELT-3′s role in stress responses.

Another possible mechanism driving DSD in this system may be related to the development of the E lineage itself. Although we have been concerned primarily with activation of the gut-specific transcriptome via ELT-2, it may be that the expanded factors in *C. elegans* fulfilled a need to directly connect the timing of cell divisions with specification. At least in *C. elegans*, the early E lineage undergoes a slowing down of its cell cycle relative to its sister lineage MS to allow the two E daughter cells to move into the embryo during gastrulation [19,135]. With stratified transcription factors activated at slightly different times, more precise timing of the cell cycle could be achieved than might be possible with only a single factor. There is evidence that connects the gut specification network with the cell cycle in *C. elegans.* Early E-specific expression of a wee1 kinase gene, *wee-1.1*, is directed by MED-1,2 [68]. Loss of *wee-1.1* by mutation causes changes in the E cell cycle without affecting differentiation, and there are other putative cell cycle regulators activated in the early E lineage [136]. Other perturbations in the E specification pathway, such as loss of *end-3* by mutation, lead to profound changes in the pattern of cell divisions arising from the E cell, causing the generation of supernumerary gut cells [24,137]. Gain-of-function mutations in *cdc-25.1*, another cell cycle regulator, can increase the number of E descendants [138,139]. These results provide evidence that the transcription factors that direct gut specification also control the cell cycle and that cell cycle factors are critical for proper patterning of the E lineage. Hence, the additional layers in the expanded network in *C. elegans* may have permitted fine-tuning of the cell cycle as compared with the simpler network in *C. angaria*.

## 12. Conclusions

Endoderm specification in *Caenorhabditis* is now established as a model for the study of DSD. The availability of genome sequences from more species and newer genetic tools will enable the testing of hypotheses for how DSD might have occurred in this system. For example, single-cell transcriptomics approaches offer a way of exhaustively identifying factors that could be important for cell specification [52,126,140]. Cell-by-cell descriptions of gene expression will be a useful resource, especially for evolutionary comparisons, and these could be complemented with perturbation studies and cross-species expression tests to identify changes in gut specification genes. With the knowledge of at least one key regulator of E specification outside of the Elegans supergroup, the stage is set in this system to perform directed functional studies aimed at understanding mechanisms by which the network might have evolved, which could be generalizable to the evolution of gene networks across many systems.

## Figures and Tables

**Table 1:** Core genes in endoderm specification in *Caenorhabditis*.

Gene	Loss-of-Function Phenotype in *C. elegans*	Loss-of-Function Phenotype in *C. briggsae* *	Loss-of-Function Phenotype in *C. angaria* *
Maternal factors			
*skn-1/Nrf*	Loss of MS, E fates [27]	Loss of MS, E fates [12]	No phenotype [9]
*pop-1/TCF*	Excess gut from MS [28]	Loss of gut [12]	Loss of gut [9]
Zygotic GATA factors			
*med-1, med-2*	Loss of MS, E fates [47]	n.d.	n/a
*end-1, end-3*	Loss of gut [13]	Loss of gut [13]	n/a
*elt-3*	No phenotype [48]	n.d.	Loss of gut [9]
*elt-7*	No phenotype; enhances *elt-2(-)* [49]	n.d.	n/a
*elt-2*	Incomplete gut differentiation [50]	n.d.	Incomplete gut differentiation [9]

* n.d.—not determined; n/a—not applicable (no orthologous genes).

## Data Availability

Not applicable.
